# Acute Lymphoblastic Leukemia Presenting as Pituitary Apoplexy: A Case Report and Review of the Literature

**DOI:** 10.1155/2021/6086756

**Published:** 2021-12-28

**Authors:** Rahul Gupta, Urmimala Bhattacharjee, K. S. Lekshmon, Shakun Chaudhary, Prashant Sharma, Aditya Jandial, Pinaki Dutta

**Affiliations:** ^1^Department of Endocrinology, Post Graduate Institute of Medical Education and Research, Chandigarh, India; ^2^Department of Clinical Hematology and Medical Oncology, Post Graduate Institute of Medical Education and Research, Chandigarh, India

## Abstract

Thrombocytopenia as a precipitating factor for pituitary apoplexy (PA) is very rare event. There are only five reported cases of PA secondary to thrombocytopenia caused by underlying haematological malignancy. Herein, we report a case of 60-year-old male presenting with acute-onset headache, bilateral vision loss, and ptosis. Computed tomography and magnetic resonance imaging revealed findings indicative of pituitary adenoma with apoplexy. He was noted to have thrombocytopenia, and bone marrow evaluation revealed precursor B-lineage CALLA-positive acute lymphoblastic leukemia. Accordingly, he was started on dexamethasone and vincristine but succumbed to *Acinetobacter baumanii*-related hospital-acquired pneumonia two weeks after initiation of chemotherapy. We performed a literature search and found five cases of pituitary apoplexy secondary to haematological malignancy-related thrombocytopenia. The usual age of presentation was in the 6^th^ to 7^th^ decade, and there was slight male preponderance. The underlying pituitary adenoma was either nonfunctioning or a prolactinoma, and in majority, the apoplexy event occurred after the diagnosis of haematological malignancy. The platelet counts at the time of PA were less than 30 × 10^9^/L in all, and the malignancy subtypes were acute or chronic myeloid leukemia and chronic lymphoid leukemia. The current case highlights the importance of careful evaluation for the cause of thrombocytopenia in a case of PA.

## 1. Introduction

Pituitary apoplexy (PA) is a clinical syndrome characterized by acute-onset headache caused by sudden hemorrhage and/or infarction. Precipitating factors are identified in only 20 to 40% of the cases [1]. Though the exact precipitating factor remains unidentified in majority, haemodynamic stress, pituitary stimulation test, coagulation disturbances, and thrombocytopenia are few of the reported risk factors [2]. Thrombocytopenia secondary to an underlying haematological malignancy as a precipitating event for apoplexy is uncommon. Here, we present a case of precursor B-cell lineage acute lymphoblastic leukaemia presenting with pituitary apoplexy. The patient provided written informed consent for allowing use of information and publication of images.

## 2. Case Presentation

**Figure 1:**
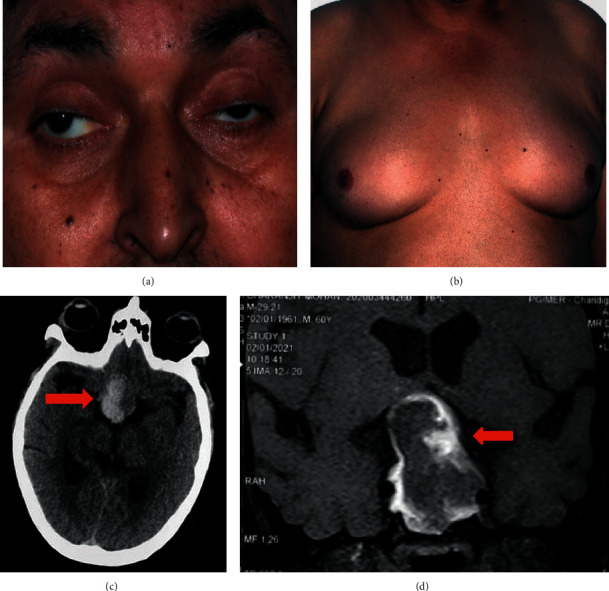
A 60-year-old male presented with sudden-onset severe headache and vision loss. (a) Bilateral ptosis and abducted right eye; (b) bilateral gynaecomastia; (c) axial NCCT head demonstrating a large sellar mass with areas of hyperdensity within suggestive of acute hemorrhage; and (d) coronal noncontrast T1-weighted MRI sellar demonstrating a 3.1 × 2.7 × 4.1 cm pituitary adenoma with peripheral T1 hyperintensity suggestive of acute bleed.

A 60-year-old male presented to the emergency department with chief complaints of sudden-onset headache, vision loss in both eyes, and drooping of eyelids for the last two days. On initial evaluation, he was haemodynamically stable, conscious, and fully oriented. Positive findings on examination included bilateral absent light perception, complete external ophthalmoplegia, bilateral ptosis, optic atrophy, and gynaecomastia (Figures 1(a) and 1(b)). A noncontrast computed tomography (NCCT) scan revealed a hyperdense sellar mass (Figure 1(c)), and contrast-enhanced magnetic resonance imaging showed a 3.1 × 2.7 × 4.1 cm sellar mass with the peripheral rim of T1 hyperintensity suggestive of adenoma with an acute to early subacute bleed (Figure 1(d)). The initial pituitary hormone profile was suggestive of secondary hypothyroidism (T4: 3.79 ug/dl (4.8–12.7); T3: 0.804 ng/ml (0.8–2); and TSH: 7.01 uIU/ml (0.27–4.2)), secondary hypogonadism (LH: 0.270 mIU/ml; testosterone 0.0868 nmol/l (9.9–27.8); and low prolactin 3.01 ng/ml (4.0–15.2)). In view of presumptive diagnosis of apoplexy, he was started on intravenous hydrocortisone before blood sample for cortisol estimation was withdrawn, but had a normal blood pressure, serum electrolytes, and plasma glucose. In view of the pituitary apoplexy score of 6 and presence of bilateral vision loss, he was planned for urgent surgical decompression [3].

**Figure 2:**
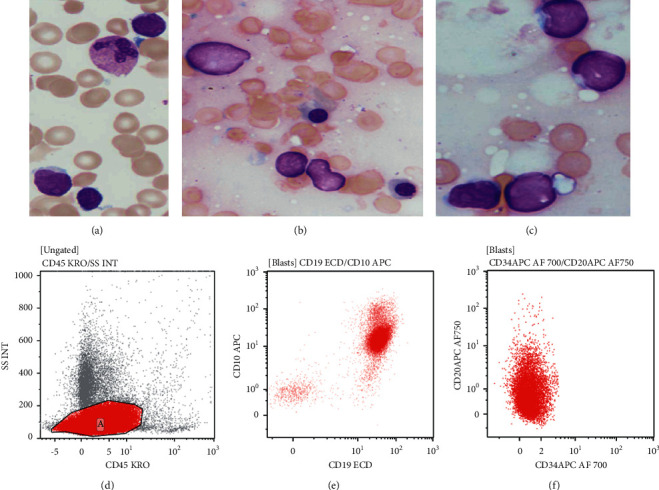
Lymphoblasts were rare in the peripheral blood (a) and comprised 70% of all cells in the bone marrow (b, c) (May–Grünwald–Giemsa stain, ×1000). On flow cytometry, they were CD45 negative to dim positive with low side scatter (d) and coexpressed CD19 and CD10 (e) as well as showed dim to negative CD20 expression (f).

He was noted to have pancytopenia on initial complete blood count (Hb: 9.8 gm/dl; TLC: 1.7 × 10^9^/L; DLC: Ne52, Ly42, Mo06%; and Plt: 23 × 10^9^/L) for which bone marrow evaluation was performed. This revealed 70% blast cells that were cytochemically myeloperoxidase negative and on flow cytometry expressed CD19, CD10, CD81, HLA-DR, CD58, and CD38 along with cytoplasmic CD79a, CD22, and nuclear TdT. The blasts were negative for T/NK cell and myeloid lineage markers, as well as for CD86, CD34, and CD123. CD20 was dim to negative. Reverse-transcriptase PCR was negative for *t* (9; 22), *t* (1; 19), and *t* (12; 21). The final hematological diagnosis was precursor B-lineage CALLA-positive acute lymphoblastic leukaemia (Figure 2).

Considering his age, poor performance status, and financial limitation, he was started on dexamethasone 10 mg/m^2^ daily and weekly vincristine 1.4 mg/m^2^. However, our patient developed *Acinetobacter baumanii*-related hospital-acquired pneumonia two weeks after initiation of chemotherapy and ultimately succumbed due to refractory septic shock.

## 3. Review of the Literature

We performed an extensive literature search in PubMed, EMBASE, and SCOPUS using the terms “pituitary, apoplexy, and thrombocytopenia,” “pituitary, apoplexy, and leukemia,” “apoplexy and thrombocytopenia,” and “apoplexy and thrombocytopenia.” We came across five cases of pituitary apoplexy associated with haematological malignancy in the English language over a period of 22 years (1996–2018, Table 1). The age at presentation was in the range of 55 years to 72 years. There was slight male preponderance with a male-to-female ratio of 3 : 2. At presentation, headache and vomiting were present in all while visual field abnormalities, opthalmoplegia, altered sensorium, and meningismus were present variably. The lag time from symptom onset to diagnosis varied from 12 hours to 6 weeks. The pituitary adenoma type was either prolactinoma or nonfunctioning, and the associated haematological malignancy was either acute or chronic myeloid leukemia or chronic lymphoid leukemia. In all except one, PA occurred after the diagnosis of haematological malignancy, and the platelet count at the time of PA was less than 30 × 10^9^/L in all. Two of the five cases were managed conservatively and the other three with transsphenoidal surgery (TSS) along with steroids. The resolution of sellar mass was seen in two cases, one managed with TSS while other conservatively (Table 1).

## 4. Discussion

We report a case of pituitary apoplexy secondary to thrombocytopenia caused by precursor B-cell acute lymphoblastic leukemia.

PA is characterized by sudden hemorrhagic infarction in a pituitary adenoma. This results in an increase in intrasellar pressure leading to a constellation of symptoms that often but not always comprise headache, nausea, vomiting, decreased vision, and diplopia. Rarely, it may lead to meningismus (secondary to extravasation of blood in subarachnoid space), hemiplegia, hypotension, and altered sensorium. True apoplexy (clinical symptoms plus radiological evidence of pituitary hemorrhage) is an uncommon event occurring in 2 to 10% of pituitary adenoma [1]. In a majority of the cases, the event is spontaneous; however, a precipitating cause is identified in 25–30% patients. Various precipitating causes reported include hypertension, hypotension, major surgery, head trauma, radiation, drugs (bromocriptne, cabergoline, antiplatelet agents, and anticoagulants), endocrine anterior pituitary stimulation test, and thrombocytopenia [4]. The two major mechanisms by which these factors cause apoplexy are either by causing acute fluctuations in blood pressure or by increasing the bleeding tendency. The pituitary gland being a neuroendocrine organ has a rich venous plexus blood supply network which predisposes it to a hemorrhagic event in response to the abovementioned stressors [4].

Thrombocytopenia leading to PA is a relatively uncommon event, and in a majority of such cases, it is either idiopathic or secondary to an underlying infection such as dengue hemorrhagic fever. To the best of our knowledge, only five cases of PA resulting from a haematological malignancy-induced thrombocytopenia have been reported so far (Table 1) [5–9]. The age of presentation was from the 5th to 7th decade, and they were either prolactinoma or nonfunctioning. In a majority of them, thrombocytopenia was a consequence of chemotherapy for the underlying malignancy and all had a platelet count of less than 30 × 10^9^/L at presentation. Our case had a nonfunctioning pituitary adenoma and presented in the 6th decade with a platelet count of 23 × 10^9^/L. However, it was unusual in that the apoplexy event was the presenting manifestation of an underlying haematological malignancy and highlights the importance of keeping this rare possibility in a case of PA with thrombocytopenia. Furthermore, the possibility of leukemic malignant cell infiltration into the pituitary adenoma cannot be ruled out in the current case, a rare but known event [7]. However, the presence of gynaecomastia suggestive of longstanding hypogonadism and optic atrophy indicates that pituitary adenoma has likely preceded the development of precursor B-cell lineage acute lymphoblastic leukaemia.

Another important issue pertains to the management of PA in the setting of thrombocytopenia. Both medical and surgical treatments are available for management of PA. The aim of surgery is to decrease the intrasellar pressure in order to decompress the optic chiasma and the nerves in cavernous sinus. Except in a scenario of worsening neurological status in whom surgery is indicated, at present, there is no clear recommendation as to the mode of therapy with the best outcome [3]. Furthermore, the presence of thrombocytopenia increases the perioperative risk of hemorrhagic complications. Though there are no clear recommendations for preoperative platelet count for neurosurgical procedures, the platelet count should be more than 100 × 10^9^/L [10]. In regard to long-term outcomes following endoscopic endonasal surgery, improvement in visual field and cranial nerve paresis is seen in 70 to 90% of the cases while hypopituitarism persists in majority [11–13]. Hence, one needs to balance the risk of perioperative hemorrhagic complications with that of uncertain long-term benefits of surgery in a case of PA with thrombocytopenia. In our patient, persistent thrombocytopenia despite platelet transfusion, intact sensorium, an improving external ophthalmoplegia, and vision in response to dexamethasone made us decide in favour of medical management. In this regard, a comprehensive evaluation by a team comprising of endocrinologists, haematologists, and neurosurgeons is of utmost importance for timely diagnosis and optimal patient outcome.

## 5. Conclusions

In a case of pituitary apoplexy associated with thrombocytopenia, a systematic evaluation for the cause of the latter is required. The selection of the management modality in such cases should balance the risk of perioperative hemorrhagic complications with that of uncertain long-term benefits of surgery, and such cases are likely to be better managed by a multidisciplinary team.

## Figures and Tables

**Table 1:** Cases of pituitary apoplexy secondary to underlying haematological malignancy.

Author	N. Wongraparut et al.	Yoshinori Maki et al.	Khaled M. Krisht et al.	L. Silberstein et al.	C. C. Kingdon et al.

Age/gender	72/F	64/F	77/M	55/M	61/M
Pituitary adenoma type	Nonfunctioning	Prolactinoma	Prolactinoma	Nonfunctioning	NA
Haematological malignancy	Acute myeloid leukaemia (subtype M5B)	Chronic myeloid leukaemia	Chronic lymphoid leukaemia	Acute myeloid leukaemia	Chronic lymphoid leukaemia
Diagnosis of apoplexy preceding haematological malignancy	No	No	No	No	Yes
Platelet count	13 × 10^9^/L	29 × 10^9^/L	NA	19 × 10^9^/L	NA
Chemotherapy	High-dose cytarabine	Cabergoline	Warfarin	NA	NA
Clinical presentation	Headache, vomiting, hypotension, b/l visual field deficit, and impairment in visual acuity	Headache, vomiting, hypotension, Rt ptosis, and Lt abducens nerve palsy	Headache, vomiting, left complete ophthalmoplegia, and diminished sensation in V1 and V2	Headache, vomiting, fever, and meningismus	Headache, fever, and altered sensorium
Lag time	12 hours	NA^*∗*^	48 hours	In hospital (day 6 of chemotherapy)	6 weeks
Hormone dysfunction	Hypothyroidism; hypoprolactinemia		Hypothyroidism; hypocortisolism	Hypothyroidism Hypocortisolism Hypogonadism	NA
Size of pituitary mass	1×1×1 cm	NA	4 × 3.7 × 3.7 cm	NA	NA
Management	TSS^#^ + steroids	TSS + steroids	TSS + steroids	Conservative	Conservative
Resolution of mass size	NA	Yes	NA	Yes	NA
Resolution of					
1. Visual field deficit	NA	NA	NA	NA	NA
2. Visual acuity deficit	Improved	Improved	NA	NA	NA
3.Ocular paresis	NA	Resolved	Improved	NA	NA

^*∗*^NA: not available; ^#^TSS: transsphenoidal surgery.

## Data Availability

The datasets generated during and/or analyzed during the current study are not publicly available but are available from the corresponding author on reasonable request.

## References

[1] Nawar R. N., AbdelMannan D., Selman W. R., Arafah B. M. (2008). Analytic review: pituitary tumor apoplexy: a review. *Journal of Intensive Care Medicine*.

[2] Briet C., Salenave S., Bonneville J.-F., Laws E. R., Chanson P. (2015). Pituitary apoplexy. *Endocrine Reviews*.

[3] Rajasekaran S., Vanderpump M., Baldeweg S. (2011). UK guidelines for the management of pituitary apoplexy. *Clinical Endocrinology*.

[4] Murad-Kejbou S., Eggenberger E. (2009). Pituitary apoplexy: evaluation, management, and prognosis. *Current Opinion in Ophthalmology*.

[5] Wongpraparut N., Pleanboonlers N., Suwattee P. (2000). Pituitary apoplexy in a patient with acute myeloid leukemia. *Pituitary*.

[6] Maki Y., Kurosaki Y., Uchino K., Ishibashi R., Chin M., Yamagata S. (2018). Pituitary apoplexy in long-term cabergoline user during thrombocytopenia due to chemotherapy for chronic myelocytic leukemia. *World Neurosurgery*.

[7] Krisht K. M., Palmer C. A., Couldwell W. T. (2013). Combined chronic lymphocytic leukemia and prolactinoma: a rare occurrence in a patient presenting with pituitary apoplexy. *Journal of Neurosurgery*.

[8] Silberstein L., Johnston C., Bhagat A., Tibi L., Harrison J. (2008). Pituitary apoplexy during induction chemotherapy for acute myeloid leukaemia. *British Journal of Haematology*.

[9] Kingdon C., Sidhu P., Cohen J. (1996). Pituitary apoplexy secondary to an underlying abscess. *Journal of Infection*.

[10] Li D., Glor T., Jones G. A. (2017). Thrombocytopenia and neurosurgery: a literature review. *World Neurosurgery*.

[11] Pangal D. J., Chesney K., Memel Z. (2020). Pituitary apoplexy case series: outcomes after endoscopic endonasal transsphenoidal surgery at a single tertiary center. *World Neurosurgery*.

[12] Zoli M., Milanese L., Faustini-Fustini M. (2017). Endoscopic endonasal surgery for pituitary apoplexy: evidence on a 75-case series from a tertiary care center. *World Neurosurgery*.

[13] Gondim J. A., de Albuquerque L. A. F., Almeida J. P. (2017). Endoscopic endonasal surgery for treatment of pituitary apoplexy: 16 years of experience in a specialized pituitary center. *World Neurosurgery*.

